# DNA-QLC: an efficient and reliable image encoding scheme for DNA storage

**DOI:** 10.1186/s12864-024-10178-5

**Published:** 2024-03-09

**Authors:** Yanfen Zheng, Ben Cao, Xiaokang Zhang, Shuang Cui, Bin Wang, Qiang Zhang

**Affiliations:** 1 School of Computer Science and Technology, Dalian University of Technology, Lingshui Street, DalianLiaoning, 116024 China; 2 The Key Laboratory of Advanced Design and Intelligent Computing, Ministry of Education, School of Software Engineering, Dalian University, Xuefu Street, DalianLiaoning, 116622 China

**Keywords:** Levenshtein code, Net information density, Combinatorial constraint, Image reconstruction

## Abstract

**Background:**

DNA storage has the advantages of large capacity, long-term stability, and low power consumption relative to other storage mediums, making it a promising new storage medium for multimedia information such as images. However, DNA storage has a low coding density and weak error correction ability.

**Results:**

To achieve more efficient DNA storage image reconstruction, we propose DNA-QLC (QRes-VAE and Levenshtein code (LC)), which uses the quantized ResNet VAE (QRes-VAE) model and LC for image compression and DNA sequence error correction, thus improving both the coding density and error correction ability. Experimental results show that the DNA-QLC encoding method can not only obtain DNA sequences that meet the combinatorial constraints, but also have a net information density that is 2.4 times higher than DNA Fountain. Furthermore, at a higher error rate (2%), DNA-QLC achieved image reconstruction with an SSIM value of 0.917.

**Conclusions:**

The results indicate that the DNA-QLC encoding scheme guarantees the efficiency and reliability of the DNA storage system and improves the application potential of DNA storage for multimedia information such as images.

## Introduction

With the rapid development of emerging technologies such as artificial intelligence, big data and blockchain, massive image data continues to emerge, and traditional storage media can no longer meet such huge storage needs. With the advantages of high storage density, long storage time, and low energy consumption, DNA storage has become one of the potential media to solve the future data storage crisis. The quality of DNA encoding and error correction methods will directly affect the cost of synthesis, sequencing and the integrity of data reading and writing, so it has attracted widespread attention from many researchers.

Early encoding methods [1–4] primarily relied on specific mapping rules to convert data into DNA sequences. To minimize the risk of errors in the DNA sequences during storage, it was essential to design DNA sequences that adhered to constraints like GC content and homopolymers. However, this approach often resulted in reduced information density. Subsequent research introduced alternative encoding methods, such as DNA Fountain [5] and the Yin-Yang codec system [6], which explored new solutions without compromising information density. However, the fountain code encoding method requires sufficient redundancy to ensure successful decoding. To address this issue, the Yin-Yang codec system has been proposed, which not only reduces decoding redundancy but also yields highly robust DNA encoding sequences. In addition, in view of the high correlation of image data, a variety of coding methods have emerged. These include coding solutions based on biological constraints [7], lossy/lossless hybrid coding schemes [8], and BO-DNA medical image coding models [9].

Although the aforementioned encoding methods have been designed to maximize information density while meeting GC content and homopolymer constraints in DNA sequences, thereby enhancing the stability and robustness of DNA storage systems, they do not entirely ensure data recovery. This is due to the high susceptibility of DNA storage systems to errors, primarily intra-sequence and inter-sequence errors. Intra-sequence errors typically occur during data writing (synthesis) and reading (sequencing) phases, leading to potential issues such as substitution, deletion, and insertion [10–12]. On the other hand, inter-sequence errors refer to the loss of sequences [13, 14]. In order to deal with this challenge, various error correction methods are designed. Currently, to address sequence loss issues, the primary approach involves adding redundant sequences [5, 15]. In the early stage, error correction codes were mainly used to correct base errors in sequences, such as RS (Reed-Solomon) codes [3, 16–18], BCH (Bose-Chaudhuri-Hocquenghem) codes [19], LDPC (low-density parity-check) codes [20, 21], HEDGES (Hash Encoded, Decoded by Greedy Exhaustive Search) error-correcting code [22], and DNA-Aeon [23]. Besides, there are also methods available that achieve error correction based on specific constraints [24] and particular rules [25]. Given the extensive exploration and research conducted by researchers in the field of encoding and error correction within DNA storage, the current systems still confront issues concerning low encoding density and relatively weak error correction capabilities.

In order to address these challenges, we propose a DNA-QLC encoding scheme. First, to improve the coding density for DNA storage systems, the scheme applies QRes-VAE (for quantized ResNet VAE) to compress images into several bitstreams. After that, the LC (Levenshtein code) is used to add check bits to the bitstreams to realize the correction of substitution, deletion, and insertion errors during DNA storage, which can solve the problem of weak error correction ability. Finally, mapping rules are used to encode bitstreams into the DNA sequence that meet combinational constraints (local GC content of 50% and homopolymers with a size of less than 2) and to avoid the occurrence of undesired motifs (GAATTC and GGC) in DNA sequences, thus improving the robustness of DNA sequences.

## Results

This study proposes DNA-QLC to enhance the performance of DNA storage systems by increasing the coding density and error correction capability. DNA-QLC was compared with previous representative works [1–6] in terms of coding results, error correction performance, and synthesis cost. The experimental results show that the DNA sequences encoded by DNA-QLC meet constraints such as local GC content and homopolymers and also avoid the occurrence of two undesired motifs, improving the robustness of DNA sequences. Moreover, given the compression ability achieved by DNA-QLC when storing images, the net information density reached by the scheme is 2.90 bits/nt, reducing the synthesis cost. In particular, at a high error rate, the SSIM value of the image before and after encoding by DNA-QLC is close to 1, indicating that the scheme has excellent error correction performance.

### Encoding result

**Fig. 1:**
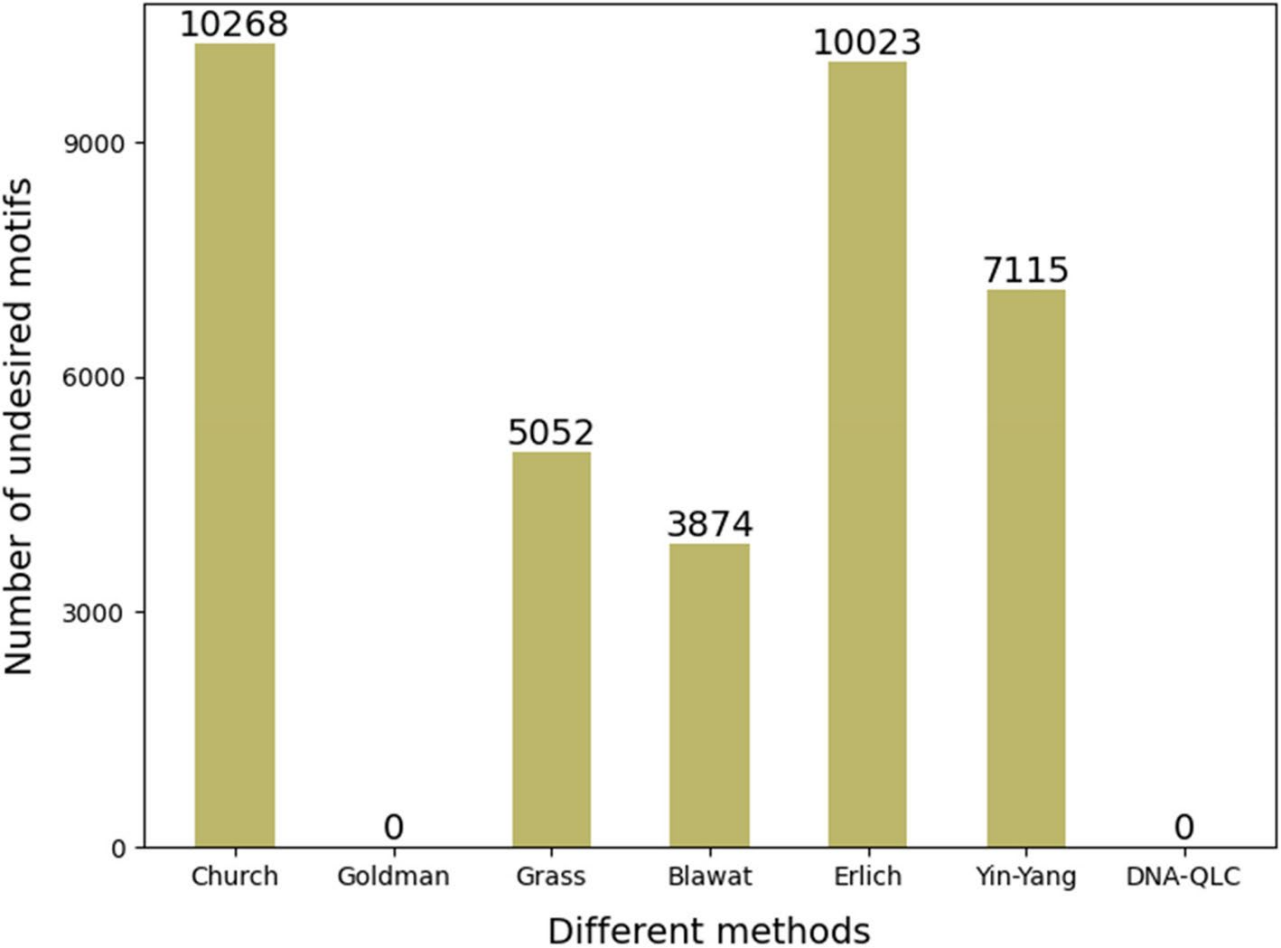
The case of undesired motifs in each encoding scheme

To prove the advantages of the DNA-QLC coding scheme, we compared it with representative coding schemes [1–6] for the same image encoding results. As shown in Table 1, in terms of net information density, DNA-QLC breaks through the limit of 2 bits per base and reaches 2.90 bits/nt, which makes it the maximum among these encoding schemes. In terms of biological constraints, the previous encoding scheme can only maintain the GC content between 40 and 60%, while DNA-QLC can control the local GC content at 50%. Similar to encoding schemes, considering the limitations of biotechnology, DNA-QLC controls the length of the homopolymer within 2 bases, which can significantly decrease the probability of errors in the process of reading and writing. In addition, the DNA-QLC encoding scheme can avoid the occurrence of two undesired motifs (GAATTC and GGC) and reduce the probability of sequence loss and the reading error rate. We used a histogram (Fig. 1) to show the situation of undesired motifs in different encoding schemes. It can be seen more intuitively that only Goldman and DNA-QLC are free from undesired motifs. In sum, besides maintaining a high information density, DNA-QLC makes the DNA sequence more highly adaptable to the process of “writing” and “reading” in the DNA storage system, improving the stability and reliability of DNA storage.

**Table 1:** Comparison of encoding schemes

Method/Reference	Error correction strategy	Number of oligos	Net information density (bits/nt)	GC content (%)	Maximum homopolymer length (nt)	Avoidance of undesired motifs
Church/ [1]	No	4064	0.94	39–61	3	No
Goldman/ [2]	Repetition	3251	1.48	39–60	1	Yes
Grass/ [3]	RS	2787	1.56	36–62	3	No
Blawat/ [4]	RS	2787	1.40	24–60	3	No
Erlich/ [5]	Fountain	2927	1.23	39–62	4	No
Yin-Yang/ [6]	RS	3125	1.36	40–60	4	No
DNA-QLC	LC	**1293**	**2.90**	**50**	2	**Yes**

**Fig. 2:**
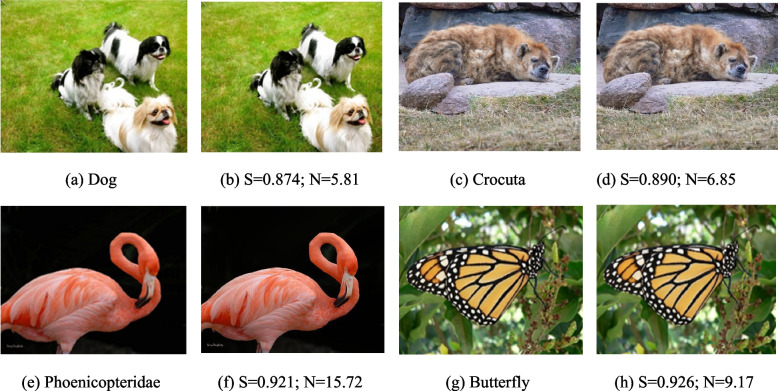
Graphic view before and after different image encoding. **a**, **c**, **e** and **g** are original image examples. **b**, **d**, **f** and **h** are based on the DNA-QLC with the results of SSIM (S) and Net information density (N, bits/nt)

To assess the reconstructed image’s quality, we measured the degree of distortion of the image and the similarity of the two images before and after encoding by SSIM [26]. SSIM is a perceptual model that correlates well with the visual experience of human eyes and considers three crucial aspects of an image: luminance, contrast, and structure. The maximum SSIM value is 1, and the minimum SSIM value is − 1. A higher SSIM value indicates a higher similarity between the original and the reconstructed image, while a lower value suggests a greater difference between the two images. The data source for SSIM value calculation was the ILSVRC2012 dataset [27], and Fig. 2 displays the results of the image comparison. The figure shows that the image obtained using DNA-QLC is visually similar to the original image and that the main objects in these images are accurately captured. For different images, DNA-QLC has a different SSIM (S) and net information density (N), but both the SSIM and the net information density are competitive. This is because the QRes-VAE model has a certain compression function that enables DNA-QLC to significantly increase the net information density while guaranteeing image quality.

### Error correction performance evaluation

**Fig. 3:**
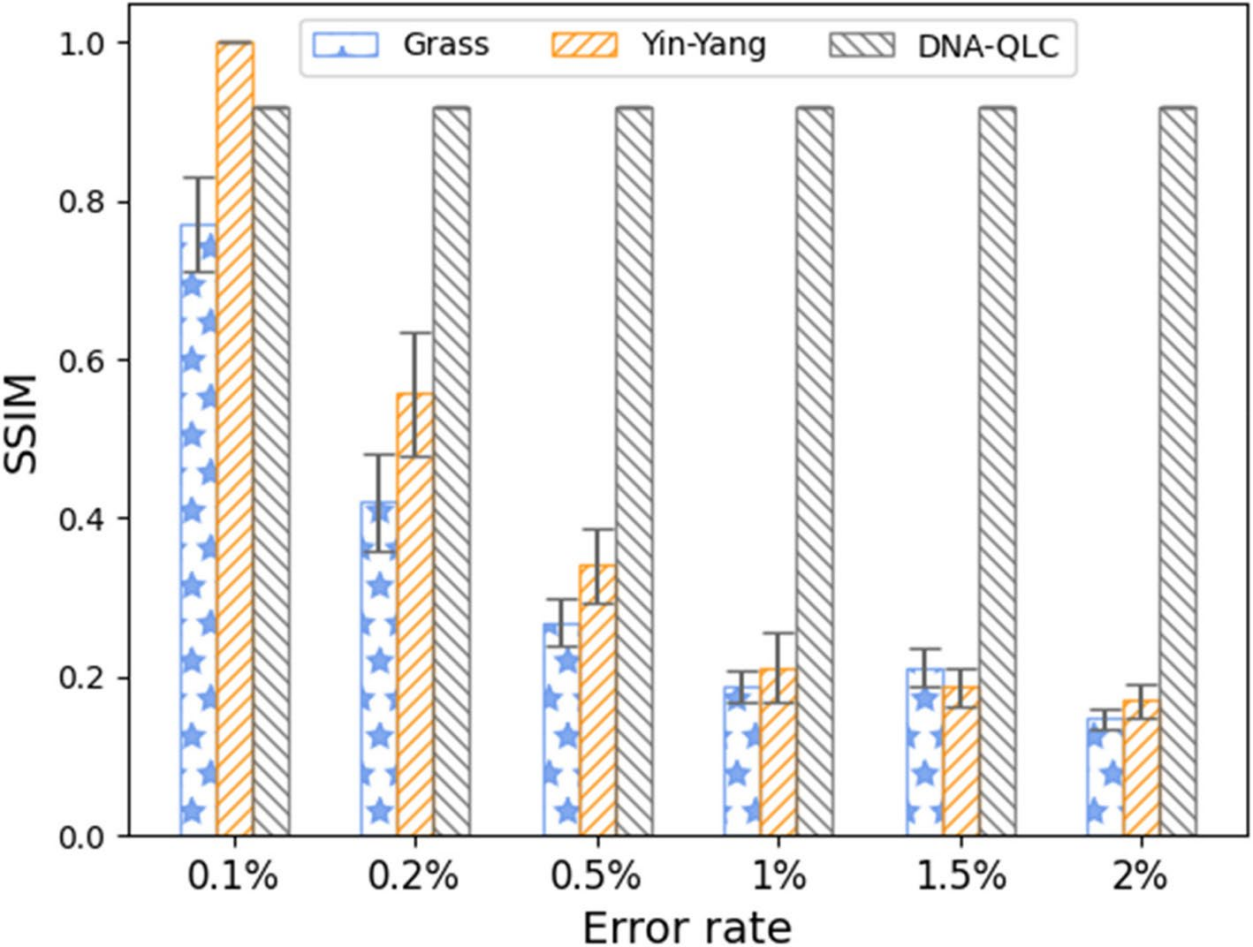
Comparison of the error correction performance of different encoding schemes under different error rates

**Fig. 4:**
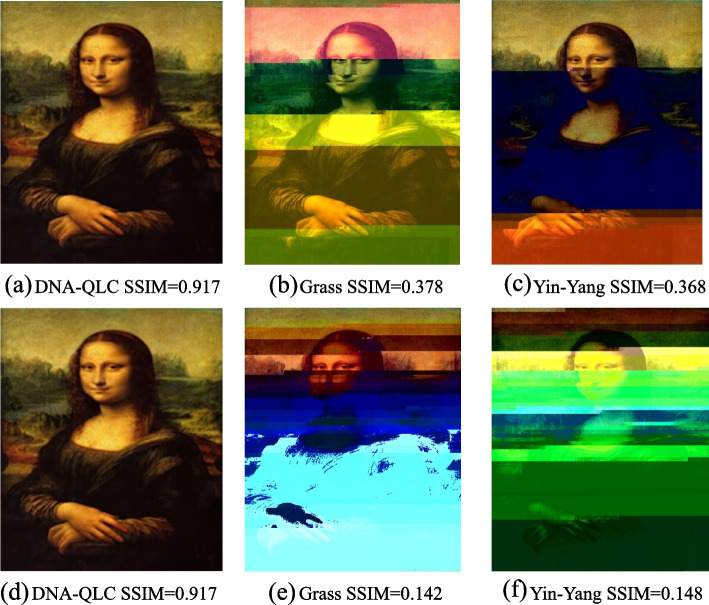
Graphic view of one example. **a**, **b** and **c** are the reconstruction of the image when the error rate of the three coding schemes is 0.5%. **e**, **f** and **g** are the reconstruction of the image when the error rate of the three coding schemes is 1.5%

To assess how effectively DNA-QLC corrects errors, we compared SSIM values with those of previous representative encoding schemes (Grass [3] and Yin-Yang [6]) under different error rates, as shown in Fig. 3. Three kinds of encoding schemes were used to encode the Mona Lisa image, and then we randomly added three types of errors: substitution, deletion, and insertion. Since substitution errors are more likely to occur than the other two types of errors, definition substitution errors account for half of the total errors when simulating errors. Here, the three encoding schemes were run 10 times under the same error rate (the error rate was maintained at 0.1–2%) to calculate the mean value and standard error. In Fig. 3, when the error rate is 0.1%, the Yin-Yang code has a higher SSIM value, which is equal to the optimal value 1, indicating that the images before and after encoding are completely consistent. However, with the increase in the error rate, the SSIM value of the encoding scheme drops sharply. As the error rate increases, the SSIM value of the Grass encoding scheme decreases clearly. However, the SSIM value of the DNA-QLC encoding scheme remains stable at 0.917 with the increase in the error rate, indicating that the scheme has a stronger error correction ability in the case of a high error rate. The shorter error bars also exclude sampling errors. Compared with the RS error correction code (Yin-Yang and Grass), DNA-QLC corrects all errors by inserting check bits into the binary stream, and it overcomes the defect that the RS error correction code has a positive correlation between the error correction performance and redundancy. Moreover, the error correction result of DNA-QLC does not fluctuate, because this scheme generates multiple DNA sequence files during the error correction process and then selects a correct sequence file from them to achieve error-free image reconstruction. Figure 4 shows the image reconstructions with error rates of 0.5% and 1.5%. DNA-QLC achieves the best visual results, further illustrating the significant advantages of the encoding scheme’s error correction capability.

### Costs of synthesizing analysis

**Fig. 5:**
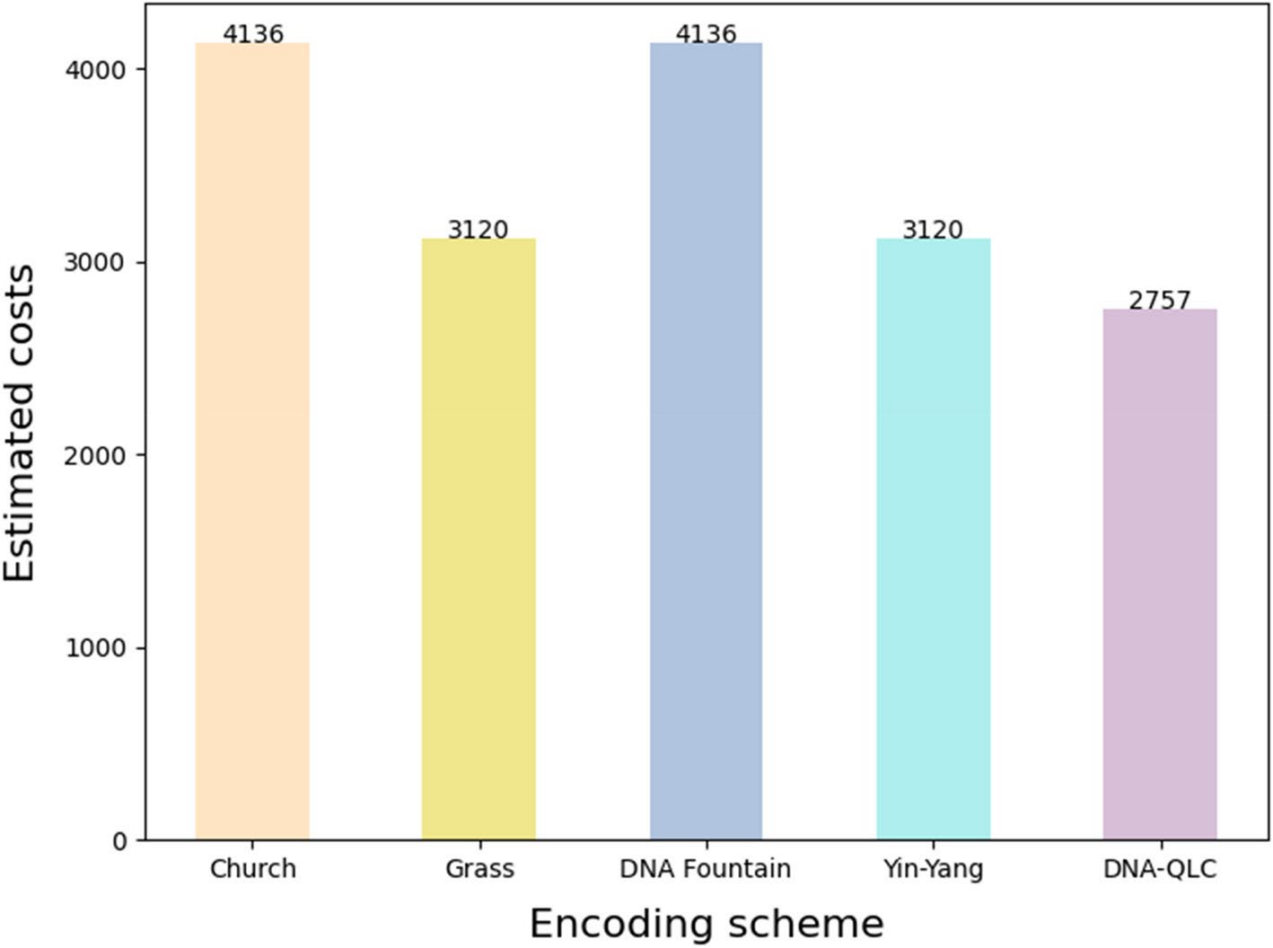
Cost evaluation of different encoding schemes

Owing to the limitations of current biotechnology, it is expensive to synthesize DNA sequences. Therefore, the encoding scheme should achieve a good error correction performance and also improve the utilization rate of the base to reduce the synthesis cost. To assess the cost, we compared DNA-QLC with four published open-source encoding schemes, including DNA Fountain [5] and Yin-Yang [6]. The Mona Lisa image data were encoded using five encoding schemes, and the oligonucleotide pool pricing table from Twist Bioscience [28] was used to approximate the synthesis cost of the encoding sequence, as shown in Fig. 5. Church’s encoding scheme requires 4064 sequences of 204 bases each, for a total of 829,056 bases to decode to obtain the Mona Lisa image. Grass’ encoding scheme requires 2787 sequences of 180 bases each. DNA Fountain requires 2927 sequences, each with 216 bases. Yin-Yang requires 3125 sequences of 184 bases each. DNA-QLC requires 1293 sequences of 208 bases each. As can be seen from Fig. 5, when a 95.2-KB image (Mona Lisa.jpg) is stored, the synthesis cost required by the DNA-QLC encoding scheme is the lowest. The DNA-QLC is 33.3% cheaper than the most widely used DNA Fountain encoding scheme and 11.6% cheaper than the latest Yin-Yang encoding scheme.

## Conclusion

In this study, aiming at the problems of low coding density and weak error-correcting ability in DNA storage, we proposed a DNA-QLC encoding scheme that uses the QRes-VAE model and the LC algorithm to compress images and correct mistakes. Comparing DNA-QLC with representative encoding schemes encoding the same image, the net information density reached by DNA-QLC is 2.90 bits/nt, 2.4 times that of DNA fountain codes (Table 1). The results in Fig. 2 show that when the input image contains significant amounts of redundant information, the net information density of DNA-QLC is 15.72 bits/nt. Clearly, the introduction of the QRes-VAE model can significantly improve the encoding density, greatly reducing the cost of DNA storage. The DNA-QLC only uses simple mapping rules to encode bitstreams into DNA sequences that meet the local GC content level of 50%, the homopolymer length of no more than 2, and no undesired motifs, effectively reducing the DNA probability of errors during DNA storage. In addition, DNA-QLC can also detect and correct multiple errors of the same type through the LC. Based on the experimental findings, we can conclude that the DNA-QLC encoding scheme can overcome the problem of the positive correlation between the error correction performance and the redundancy of other encoding schemes. And with the increase of the error rate, the image SSIM value will not decrease. In addition, when the error rate is high, the DNA-QLC encoding scheme can still maintain the integrity and clarity of the image, and they do not cause serious distortion or the failure to recognize the main object (Fig. 4).

Although DNA-QLC has high encoding and error correction performance, DNA-QLC has a defect in correcting the substitution error, that is, it can only correct the errors of purine mutation to pyrimidine or pyrimidine mutation to purine. We will attempt to resolve this issue in future research work as well as study the molecular characteristics of DNA, construct a DNA storage self-error correction model based on deep learning, reduce the overhead of unnecessary error correction redundancy, further improve the net information density and capacity of DNA storage, reduce its cost, and promote DNA storage practical applications in storing cold data.

## Methods

**Fig. 6:**
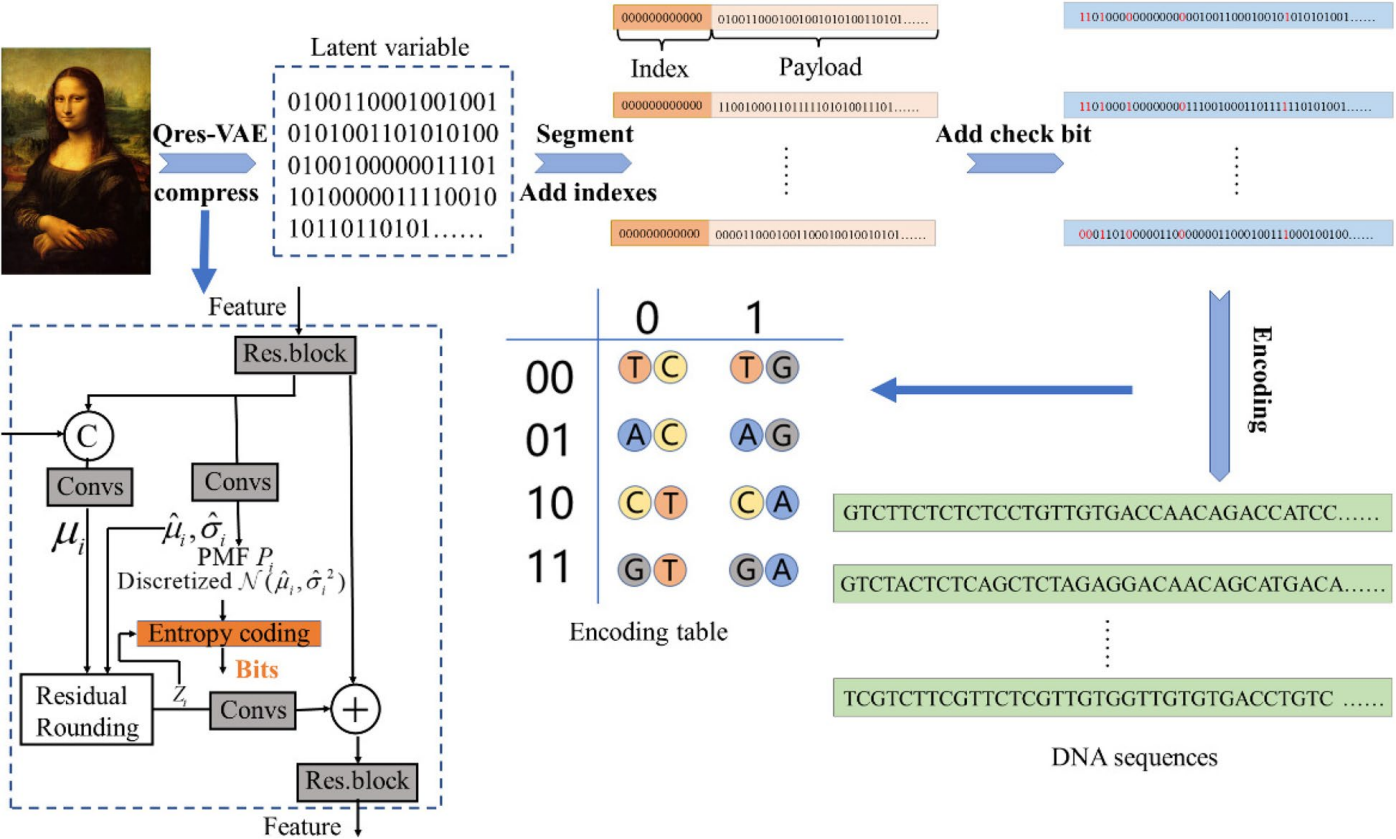
Flowchart of the DNA-QLC encoding scheme

To improve the coding density and error correction performance in the DNA storage system, we propose a DNA-QLC encoding scheme, which is primarily divided into QRes-VAE compression model and LC encoding algorithm. First, the input image is compressed using the QRes-VAE model to obtain compressed binary data, which are segmented and indexed. Then, the LC is used to add check bits to the binary data. Finally, the bitstreams are mapped to DNA sequences that comply with the combinatorial constraints through mapping rules. The flowchart and pseudocode of DNA-QLC are shown in Fig. 6 and Algorithm.

**Figure:**
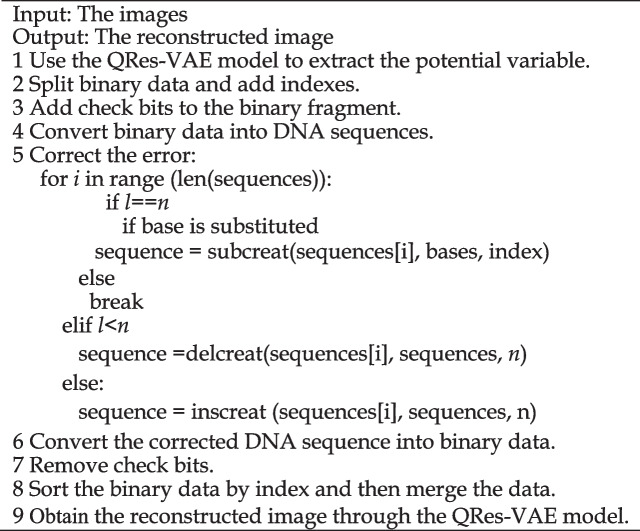
**Algorithm 1.** The pseudocode of DNA-QLC

### Image compression with QRes-VAE

Compression technology is critical for DNA storage. An efficient compression method can improve the utilization rate of the base while ensuring the integrity of data, increasing the coding density, and reducing the synthesis cost. However, the performance of current compression techniques in DNA storage is lacking, such as Huffman coding [2] and discrete wavelet decomposition [7]. In this paper, images are compressed by the QRes-VAE model, and the compression process of this model [29] is described in detail in this subsection.

#### Network architecture

The QRes-VAE model is based on ResNet (residual network) VAE [30], using quantization-aware posteriors and priors to redesign latent variables. It consists of a bottom-up path and a top-down path. When an image is inputted, the bottom-up path produces some deterministic features, which are then transmitted to the top-down path for inference, after which the image is reconstructed by up-sampling.

#### Loss function

The loss function of the QRes-VAE model [29] is shown as follows:

1 $$\begin{array}{c}\mathcal{L}={D}_{{\text{KL}}}\left({q}_{Z\left|x\right.}\Vert {p}_{z}\right)+{\mathbb{E}}_{{q}_{Z\left|x\right.}}\left[{\text{log}}\frac{1}{{P}_{\left.X\right|Z}\left(\left.x\right| Z\right)}\right],\\ ={\mathbb{E}}_{{q}_{Z\left|x\right.}}\left[\sum\limits_{i=l}^{N}{\text{log}}\frac{1}{{P}_{i} \left({Z}_{i}\left|{Z}_{<i}\right.\right)}+\lambda \cdot d\left(x,\widehat{x}\right)\right]+{\text{constant}},\end{array}$$

where $Z\sim {q}_{Z\left|x\right.}$ represents the estimation of samples extracted in each training step, $\lambda$ is the hyperparameter that can be set manually, $x$ is the input image, $\overline{x }$ is the reconstruction image, and $d\left(x,\overline{x }\right)$ is the mean square error of the input image and reconstruction image.

#### Compression

First, features are extracted from the input image and quantized into *N* potential variables (${Z}_{1},{Z}_{2},\dots ,{Z}_{N}$). Then, potential variables are encoded into bits by using the probability quality function (PMF) [31]. Finally, entropy encoding is performed by the range-based asymmetric numeral systems (rANS) [32]. The quantization formula [29] during compression is as follows:

2 $$z\leftarrow{\widehat v}_i+\lfloor v_i-{\widehat v}_i\rceil,$$

where $\lfloor\cdot\rceil$ is the nearest integer function, and ${v}_{i}$ is quantified as its nearest neighbor in the set $\left\{{\widehat{v}}_{i}+n \left| n\in {\mathbb{Z}}\right.\right\}$, denoted by ${Z}_{i}$.

The formula for the PMF ${P}_{i}\left(\cdot \right)$ [29] is given below.

3 $${P}_{i}\left(n\right)\triangleq {p}_{i}\left({\widehat{v}}_{i}+n\left| {Z}_{<i}\right.\right), n\in {\mathbb{Z}}$$

Decompression is the inverse process of compression. It first uses rANS to decode each bitstream and transform ${Z}_{i}$ using convolution layers before adding it to the feature. Finally, it obtains the reconstructed image through the final up-sampling layer in the top-down decoder. The QRes-VAE model is used to compress images so that a small number of DNA sequences can be used to store image information. This approach improves the coding density of the DNA storage system and reduces the cost.

### Levenshtein code algorithm

In the process of DNA synthesis, PCR amplification and DNA sequencing, substitution, deletion and insertion errors are easy to occur. Previous studies have reported that chemistry synthesis and second-generation sequencing result in an error rate of about 1% per base [15] and that third-generation sequencing has an error rate of up to 10% [33]. Moreover, the error rate of DNA sequences varies among different motifs. DNA sequences with homopolymers and abnormal GC content are difficult to synthesize [6], thus generally having a higher error rate during the synthesize process. Therefore, constraints are crucial to avoiding errors in DNA storage, and more new constraints are being widely explored and studied. For example, DNA sequences with restriction sites were easily cleaved by restriction enzymes (“GAATTC” for “EcoRI”) during in vivo storage, leading to information loss [34]. For the replication process, local GC content balance was explored to improve the success rate of PCR amplification technology [35]. During the sequencing process, the Illumina sequencing platform had a higher error probability for DNA sequences containing “GGC” fragments [36].

Most current encoding schemes can only meet the two basic constraints of a global GC content and homopolymer and cannot satisfy the abovementioned new constraints. Therefore, a novel mapping rules was designed, whose central idea is to map three binary numbers to two bases (e.g., 000 $\to$ TC, 001 $\to$ TG, 010 $\to$ AC, 011 $\to$ AG, 100 $\to$ CT, 101 $\to$ CA, 110 $\to$ GT, 111 $\to$ GA). The mapping rules, which sets purine and pyrimidine in series, can well control the local GC content to 50% and simultaneously set keep the maximum limit of homopolymers 2 and exclude the occurrence of two undesired motifs (GAATTC and GGC). However, constraint encoding can only reduce the error probability, but can not completely avoid it. To further ensure the read–write integrity of data, LC [37] is used to correct substitution, deletion, and insertion errors that occur within the sequence.

#### LC

This is a binary algebraic code whose binary codeword of length $n$ satisfies Eq. (4) [37].

4 $$L\left(m,r,U\right)=\left\{x\in {\left\{\mathrm{0,1}\right\}}^{m}:\sum\limits_{k=1}^{m}{x}_{k}*k\equiv r\ {\text{mod}}\ U\right\}$$

For any integer $U\ge 2m$, $0\le r\le U-1$, in this study, let $r=0$ and $U=2m$. In sum, an $l$ bits binary sequence is processed into a codeword of length $m$, and the conditions below should be satisfied.

5 $$\sum\limits_{k=1}^{m}{x}_{k}*k\equiv 0\ \mathrm{mod\ }2m$$

The central idea of the LC is actually to insert parity bits at 2^i^-th positions to ensure that the codeword has a desired syndrome. Note that the last position is always a parity bit and that the second-to-last position is the message bit. When the length of the binary data is $l$, the length of the codeword processed by the is $m$, and the calculation equation is as below.

6 $$l=m^{\prime}-\lceil{{\text{log}}}_{2 }m^{\prime}\rceil-1$$

7 $$m={\text{min}}\ m^{\prime}$$

#### Example

The binary data are 101,000,011,110, and the length $l=12$, which is calculated by Eqs. (6) and (7) to obtain $U=36$. To meet the conditions of Eq. (5), we must use an additional 6 check bits.

Calculate the syndrome of the bits using $\sum\limits_{k=1}^{m}{x}_{k}*k=3+6+12+13+14+15=63$. Through $2U-63$, the syndrome can be calculated to be equal to 9, we can convert it into binary (001001) and obtain 6 check bits, then we insert that value into the binary data stream and obtain the code word 101,001,010,001,111,000 processed by LC. Then, according to the mapping rules, the code word 101,001,010,001,111,000 can be encoded into the DNA sequence CATGACTGGATC, which satisfies the local GC content of 50%, the homopolymer length not exceeding 2, and no GAATTC and GGC two undesired motifs. In addition, if the sequence has substitution, deletion, and insertion errors during the DNA storage process, the added syndrome can be used to correct the sequence to obtain the correct original information.

## Data Availability

The data and code underlying this article are available in https://github.com/Larissa-11/DNA-QLC.
